# Intubation first-pass success in a high performing pre-hospital critical care system is not associated with 30-day mortality: a registry study of 4496 intubation attempts

**DOI:** 10.1186/s13049-022-01049-7

**Published:** 2022-11-21

**Authors:** Harry Ljungqvist, Jussi Pirneskoski, Anssi Saviluoto, Piritta Setälä, Miretta Tommila, Jouni Nurmi

**Affiliations:** 1 University of Helsinki, Helsinki, Finland; 2 Department of Emergency Medicine and Services, Helsinki University Hospital and University of Helsinki, Helsinki, Finland; 3 Centre for Prehospital Emergency Care, Helicopter Emergency Medical Services, Tampere University Hospital, Tampere, Finland; 4 Department of Perioperative Services, Intensive Care Medicine and Pain Management, Turku University Hospital and University of Turku, Turku, Finland

**Keywords:** Air ambulances, Emergency medical services, Critical care, Airway management, Rapid sequence induction and intubation, First-pass success

## Abstract

**Background:**

Lower intubation first-pass success (FPS) rate is associated with physiological deterioration, and FPS is widely used as a quality indicator of the airway management of a critically ill patient. However, data on FPS’s association with survival is limited. We aimed to investigate if the FPS rate is associated with 30-day mortality or physiological complications in a pre-hospital setting. Furthermore, we wanted to describe the FPS rate in Finnish helicopter emergency medical services.

**Methods:**

This was a retrospective observational study. Data on drug-facilitated intubation attempts by helicopter emergency medical services were gathered from a national database and analysed. Multivariate logistic regression, including known prognostic factors, was performed to assess the association between FPS and 30-day mortality, collected from population registry data.

**Results:**

Of 4496 intubation attempts, 4082 (91%) succeeded on the first attempt. The mortality rates in FPS and non-FPS patients were 34% and 38% (*P* = 0.21), respectively. The adjusted odds ratio of FPS for 30-day mortality was 0.88 (95% CI 0.66–1.16). Hypoxia after intubation and at the time of handover was more frequent in the non-FPS group (12% vs. 5%, *P* < 0.001, and 5% vs. 3%, *P* = 0.01, respectively), but no significant differences were observed regarding other complications.

**Conclusion:**

FPS is not associated with 30-day mortality in pre-hospital critical care delivered by advanced providers. It should therefore be seen more as a process quality indicator instead of a risk factor of poor outcome, at least considering the current limitations of the parameter.

## Background

Tracheal intubation remains the gold standard in pre-hospital advanced airway management (PHAAM) [1]. Excluding patients in cardiac arrest, PHAAM is usually performed as a rapid sequence induction (RSI) and intubation, a critical intervention during pre-hospital emergency care requiring advanced skills in equipment and pharmaceutical use, team resources management and treating possible adverse effects [2–4]. Critically ill patients represent the highest-risk patients to intubate; complications such as hypoxia, hypotension, and cardiac arrest are frequent [5–7]. The deterioration of physiology is further exacerbated during airway management when intubation requires more than one attempt [8, 9]. Hence, RSI protocols emphasise the importance of achieving successful endotracheal intubation on the first attempt to reduce the incidence of adverse effects [2].

Thus, the first-pass success (FPS) rate has come to reflect the quality and safety of the intubation procedure in airway management. High-performing pre-hospital systems have displayed an impressive increase in the FPS rate in recent years [10–12]. However, the FPS rate has been criticised as RSI’s main quality indicator, as even if complications increase with multiple attempts, the association between FPS and mortality is not established [8, 13, 14].

We aimed to evaluate the FPS rate, as a measurement of the quality of care during pre-hospital RSI, by studying its association with complications and mortality. Furthermore, we wanted to report the success rate of pre-hospital RSI in Finnish helicopter emergency medical services (HEMS). Although FPS represents only a small portion of PHAAM’s complex process, it still incorporates many aspects of high-quality performance. Thus, FPS might function as a valuable quality indicator and predictor of mortality.

## Methods

### Study design

We performed a retrospective observational study to assess the independent association between FPS and 30-day mortality. Helsinki University Hospital’s ethical committee and all registry data owners approved the study protocol. Data access was granted by all hospitals responsible for HEMS (Oulu University Hospital 200/2019 2.7.2019, Helsinki University Hospital HUS/280/2019 9.7.2019, Turku University Hospital J30/19 4.8.2019, Hospital District of Lapland 32/2019 22.8.2019, Kuopio University Hospital RPL 102/2019 22.8.2019 and Tampere University Hospital RTL-R19580 2.9.2019), the Finnish Institute for Health and Welfare (THL/2231/5.05.00/2019) and the Digital and Population Data Services Agency (VRK/5613/2019-3). The Strengthening the Reporting of Observational Studies in Epidemiology (STROBE) [15] statement is followed in reporting this study.

### Setting

This study was performed in Finnish HEMS, consisting of five physician-staffed units and one unit staffed by advanced paramedics serving the sparsely populated northern part of Finland. HEMS provides advanced level pre-hospital critical care and rapid helicopter transport as necessary. The same teams operate by rapid response cars when weather conditions do not allow helicopter use or the patient is near the HEMS base. HEMS is dispatched by the emergency response centres, according to predefined dispatching criteria, or later upon request by on-scene emergency medical services. The largest patient groups treated are major trauma, out-of-hospital cardiac arrest (OHCA) and unconsciousness due to, for example, intracranial haemorrhage or poisoning [16].

Physicians in Finnish HEMS are mostly consultants or final-year residents of anaesthesia and intensive care medicine, and the turnover is low [17]. The proportion of worktime in HEMS and hospitals varies substantially among physicians. The advanced level paramedics have extensive education and experience in pre-hospital critical care.

No national protocol for PHAAM exists in Finland. Various equipment, including direct and video laryngoscopes, bougies and pre-inserted stylets, were available. For inducing anaesthesia, the units had anaesthetics, neuromuscular blocking agents (NMBA) and opioids at their disposal, except for the paramedic staffed unit in Lapland that did not have NMBA. One base implemented a strict pre-hospital anaesthesia protocol, including the selection of anaesthetic drugs, use of C-MAC videolaryngoscopy and Frova introducer as a first-line intubation strategy [11]. During the study period some of the other bases also implemented local protocols. However, the consistency in the laryngoscopy strategies has not been reported. Extensive monitoring was possible at all units, including waveform capnography and invasive blood pressure monitoring.

### Participants and data sources

We included all patients with attempted drug-facilitated intubation in Finnish HEMS between January 2014 and August 2019, regardless of the indication of airway management. Patients with missing data on FPS were excluded. The data were collected from the national HEMS quality database. The physician or advanced paramedic overseeing the HEMS mission is responsible for entering detailed structured data into the database after each mission. The database has been in use since 2012, and the amount of missing data is low. The database has been described and the data quality reported [16, 18]. The database follows the international recommendations for data collection from physician-provided pre-hospital critical care and PHAAM [19, 20].

Survival was followed for 30 days using the National Population Registry data. Patients were identified by the unique personal identification number, addressed to all residents in Finland.

### Variables

The FPS was the exposure studied. The primary outcome was 30-day mortality; secondary outcomes were reported complications including hypoxia, hypotension, and death before reaching the hospital. Hypoxia is defined as an oxygen saturation below 90% and hypotension is defined as a systolic blood pressure below 90 mmHg, these complications was not to be present before airway intervention and had to be recorded during or immediately after airway management. These definitions of complications followed the PHAAM data collection recommendations [19]. Known variables associated with mortality in HEMS patients but not all directly associated with the PHAAM process were collected for multivariable analysis. These variables included age, sex, Glasgow Coma Score (GCS) and the first vital signs measured when the HEMS unit encountered the patient, as well as patient category and the time from the alarm to reaching the patient. These variables were chosen based on existing literature, statistical reasoning, and availability [21–24]. Patient categories were originally classified in the database according to the data collection recommendations, but combined into five groups due to the small number of patients in some groups [25].

### Statistical methods

For the primary outcome (30-day mortality), we performed a multivariable logistic regression analysis using age, sex, GCS, heart rate, systolic blood pressure, oxygen saturation, patient category, time from the alarm to reaching the patient and the FPS as predictors. No stepwise methods were used when constructing the model, all variables were entered into the model simultaneously. For 79 patients sedated before being encountered by the HEMS unit, a GCS of three was used. Only cases with all included covariates recorded where included in the multivariate regression analysis. The results of the regression analysis are reported as odds ratio (OR) with 95% confidence interval (95% CI). The distribution of our data was assessed visually with virtually all data being skewed. Descriptive data are therefore reported as number (percentages) for proportions and medians (interquartile range) for continuous variables. The Mann-Whitney U test was used to compare continuous data and the Chi2 test was used to compare categorical data. A *P*-value < 0.05 was deemed to be statistically significant. Missing data were excluded from the analyses.

All available data were used, and no power analysis was performed beforehand. A post-hoc power calculation was performed to assess the risk of type II error. All statistical analyses were done using SPSS Statistics for Mac, version 27 (IBM Corp., Armonk, NY, USA).

## Results

**Fig. 1:**
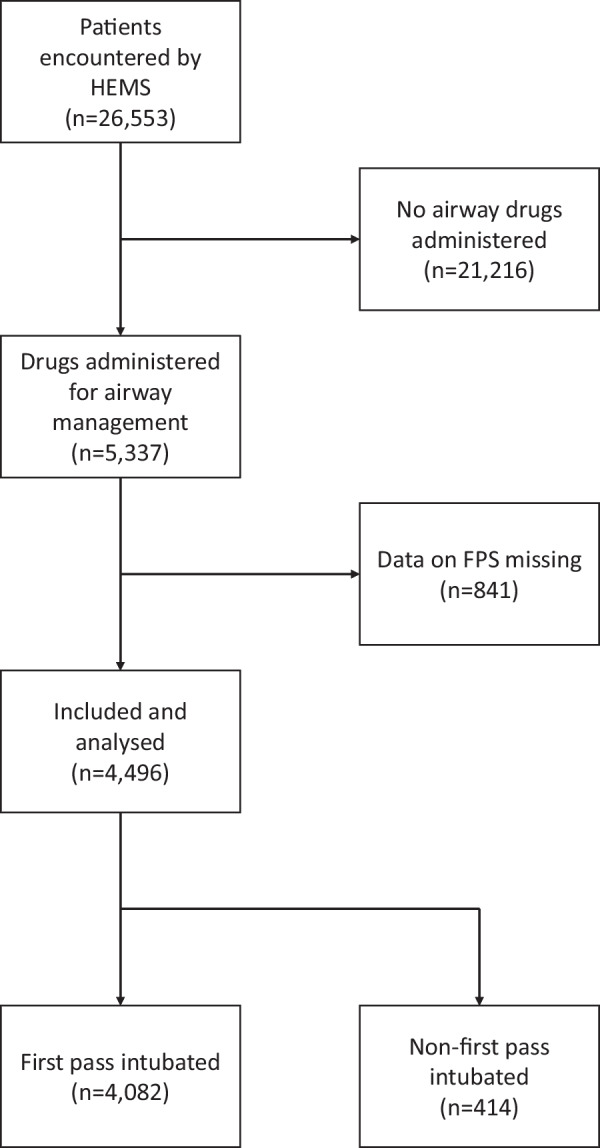
Patient selection flow chart

During the study, the HEMS teams attempted 4496 drug-facilitated intubations; all were included in the study. Figure 1 shows the flow chart of patient selection.

The median patient age was 59 (40–71), and 3476 (65%) were male. The distribution among patient categories was 1638 (31%) neurological, 1242 (23%) OHCA, 1236 (23%) trauma, 695 (13%) intoxication and 526 (10%) other. OHCA indicates anaesthesia provided as part of post-resuscitation care. Table 1 shows the baseline patient characteristics according to FPS.

**Table 1:** Baseline patient characteristics stratified by first-pass success at intubation

	FPS *n* = 4082	Non-FPS *n* = 414
*Patient characteristics*		
Age	59 (40–71)	61 (44–70)
Sex; male	2646 (65)	287 (69)
*Patient category*		
Trauma	934 (23)	76 (18)
OHCA	935 (23)	120 (29)
Neurological	1284 (31)	121 (29)
Intoxication	554 (14)	57 (14)
Other	375 (9)	40 (10)
*Vital signs at time of patient encounter*		
Respiratory rate (1/min)	16 (12–22)	18 (12–25)
Oxygen saturation (%)	96 (91–99)	95 (89–98)
Heart rate (1/min)	94 (75–114)	100 (80–119)
Systolic blood pressure (mmHg)	133 (110–162)	132 (105–160)
GCS	3 (3–6)	3 (3–6)
*Operational characteristics*		
Time from alarm to patient contact (min)	23 (17–35)	25 (16–39)
Transport duration (min)	26 (16–40)	30 (17–45)
Transported by helicopter	600 (15)	79 (19)

Presented as median (25–75th percentile) or *n* (%). Respiratory rate, oxygen saturation, heart rate, systolic blood pressure and GCS at patient encounter were available for 3469 (77%), 3895 (87%), 4099 (91%), 3999 (89%) and 4496 (100%), respectively. Transport duration could not be calculated for 449 (10%) cases because of missing timestamps. FPS, intubation first-pass success; OHCA, out-of-hospital cardiac arrest (anaesthesia provided as part of post-resuscitation care); GCS, Glasgow Coma Scale

A total of 4082 patients were intubated on the first attempt (FPS rate 91%); the overall success rate was 99.7%. The FPS group received more often neuromuscular blockade and had a shorter on-scene time (Table 2).

**Table 2:** Drugs used to facilitate intubation and on scene time stratified by first-pass success at intubation

	FPS *n* = 4082	Non-FPS *n* = 414	*P*-value
Neuromuscular blockade	3708 (91)	333 (80)	< 0.001
Sedative agent	3880 (95)	385 (93)	0.071
Analgesia	3190 (78)	338 (82)	0.099
On-scene time (min)	33 (23–43)	40 (29–52)	< 0.001

Data presented as n (%) or median (25–75th percentile). On-scene time could not be calculated due to missing time stamps for 273 (6%) cases. No missing data for other variables. FPS, intubation first-pass success

**Fig. 2:**
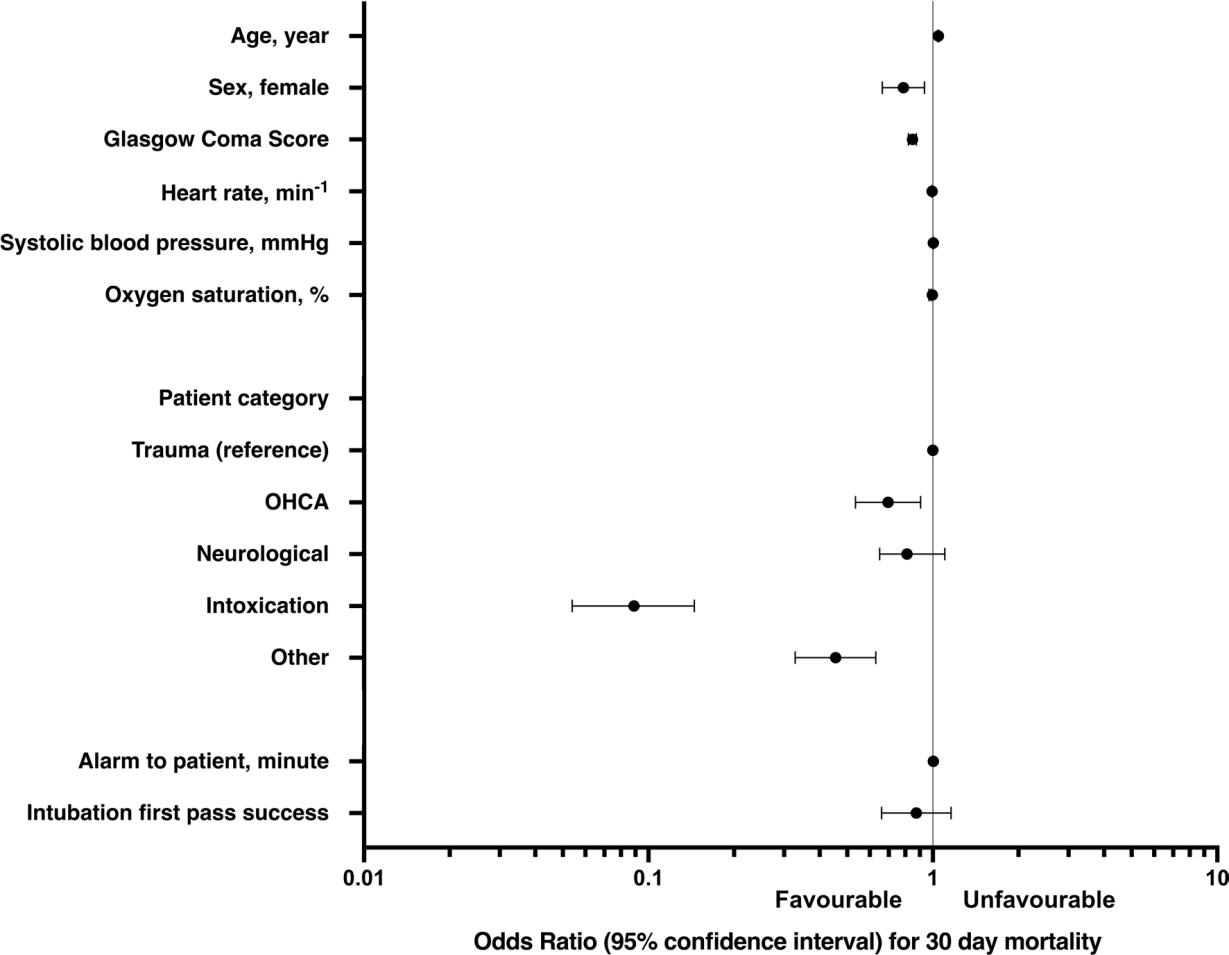
Multivariate logistic regression model for 30-day mortality of 3683 patients undergoing pre-hospital anaesthesia that had data available on all included covariates. OHCA, out-of-hospital cardiac arrest (anaesthesia provided as part of post-resuscitation care)

Complete data on 30-day mortality was available for 4355 (97%) patients. Out of these patients, 1504 (34%) died within 30 days of pre-hospital care. No significant difference was observed in the mortality rate between FPS and non-FPS patients (34% vs. 38%, *P* = 0.21). Furthermore, after adjusting for potential confounders, there was no significant independent relationship between FPS and 30-day mortality (Fig. 2). All neccesary covariates were available for 3683 patients and thus were included in the multivariate logistic regression analysis. The adjusted odds ratio of FPS for 30-day mortality was 0.88 (95% CI 0.66–1.16). Based on the post hoc power analysis, the sample size has 80% power (alpha 0.05) to detect a mortality rate difference of 7% (33% vs. 40%), with an actual allocation of subjects to FPS and non-FPS groups.

Table 3 presents the secondary outcomes. FPS and non-FPS groups differed in the hypoxia rate after intubation and at patient handover, whereas no difference was observed regarding other complications.

**Table 3:** Physiological complications according to intubation first-pass success

	FPS *n* = 4082	Non-FPS *n* = 414	*P*-value
Hypoxia post-intubation	160 (5)	32 (12)	< 0.001
Hypotension post-intubation	366 (12)	44 (16)	0.072
Hypoxia at time of handover	108 (3)	20 (5)	0.010
Hypotension at time of handover	202 (5)	24 (6)	0.398
Survival to hospital	3879 (97)	394 (96)	0.154

Data presented as n (percentage). Data for hypoxia post-intubation, hypotension post-intubation, hypoxia at handover and hypotension at handover were available for 3298 (73%), 3312 (74%), 4120 (92%) and 4154 (92%) patients, respectively. Survival to hospital was recorded for 4396 (98%) patients. FPS, intubation first-pass success

## Discussion

In this study, we found a high FPS rate by highly skilled pre-hospital critical care providers and no association between FPS and 30-day mortality. However, a failed first intubation attempt was associated with hypoxia immediately following intubation and at patient handover to the hospital.

In high-performing pre-hospital critical care, high FPS rates (84.5%) of endotracheal intubation are achieved rapidly (25 s) and with relatively short on-scene times (25 min) [10]. A prior study in our system showed that when a standardised protocol is implemented for RSI, the FPS rate can be as high as 98.2% [11]. Multiple intubation attempts have clearly shown to increase the risk of adverse events. It thus seems intuitive that FPS is vital in the intubation procedure [8, 9, 26]. The FPS rate was also recently presented as one of PHAAM’s core quality indicators [27–29]. In the current study of a national HEMS system, the FPS rate was high and comparable with previous findings [10].

Many factors influence the FPS rate, including environmental, patient, provider, and system-related factors [30–32]. In our study, patients intubated with more than one attempt received NMBA less frequently, which might be partially explained by the fact that the northernmost HEMS unit is paramedic-staffed and does not use NMBA, possibly affecting FPS [33, 34].

However, despite the established position of the FPS rate, the association between FPS and mortality remains unclear. Furthermore, high complication rates are still recorded despite FPS, especially in critically ill patients [14, 35]. In fact, a study regarding emergency department patients found no association between multiple attempts at intubation in the emergency department and in-hospital survival, although a higher rate of adverse events was noted [13]. Supporting this, our study found the same lack of association between FPS and mortality while revealing a connection with hypoxia.

If the FPS rate is high, it might reflect that the pre-hospital critical care and intubation are also performed to a high standard. If so, this might diminish the influence of the FPS rate per se. We suggest that over-emphasising the FPS value in single cases in a high-performing HEMS setting might not be meaningful. FPS should instead be used primarily as a system-level quality marker. Taking care of overall patient safety rather than focusing solely on the number of intubation attempts is the most crucial concern, especially as the specific conditions for aborting the first intubation attempt remain unstandardised [6, 36]. Even in a recent consensus paper on PHAAM quality indicators, the threshold to terminate an attempt remains undefined and will need further clarification to improve future studies and their interpretations. One solution could be combining FPS with avoiding adverse events for a more clinically relevant variable, as used in some recent studies [35, 37, 38]. Our results also displayed a significant difference in on-scene time between FPS and non-FPS groups. Possibly reflecting more complex cases in the non-FPS group resulting in overall delayed on-scene times or more difficult airways requiring prolonged management.

To our knowledge, this is the first nationwide study reporting the association of the intubation FPS rate and mortality in HEMS. Although the number of PHAAM included in this study is high, it is still underpowered to detect a smaller difference in mortality, according to the post hoc calculations. The post hoc power calculation shows the power is sufficient to determine a mortality difference approximately twice as large as observed in the study. If a difference in the mortality rate exists, as observed in the current study, over 20,000 patients would be needed to achieve adequate statistical power. This corresponds to pre-hospital intubations of multiple decades in our country. Thus, combining data from different countries in future studies is necessary. Furthermore, if such a high number of procedures demonstrate the differences in mortality, it is debatable whether the extent of the effect is clinically significant.

Our study has several limitations. Firstly, this retrospective registry study increases susceptibility to different biases, as reporting airway management is prone to subjective factors, the database is unvalidated, and no external referee is used during pre-hospital care [39]. Secondly, when studying a HEMS intervention in a pre-hospital setting, several confounding factors are challenging to take in to account for the statistical analysis. Examples of these factors include the quality of care before HEMS arrival, the quality of in-hospital care, emergency medical response time and the time from the event to dispatch. We also had some amount of missing data regarding first pass success and mortality, which may introduce bias of its own. The intervention studied was not standardised between units, as no national standard operating procedure for RSI was implemented. However, a standardised data template for advanced airway management is implemented in the HEMS database, and the data are uniform [20].

We recognise PHAAM’s complexity and that many factors can influence patient outcomes [40]. Also, 30-day mortality can be considered a crude outcome quality measure. The quality of life after the pre-hospital event could be regarded as a more refined outcome, especially given the result of the FPS rate on hypoxia shown in this study [22, 41]. Our study did not include data on functional outcomes; thus, we could not examine whether the FPS rate impacts this.

## Conclusions

In conclusion, the FPS rate was not associated with 30-day mortality in nationwide Finnish HEMS with an overall high FPS rate. We suggest the FPS rate serves better as a quality indicator describing the complex entity of the PHAAM process rather than just a mortality outcome predictor, at least considering the current limitations of the parameter. However, care should be taken when assessing generalisability, as HEMS systems and patient demographics might differ substantially. The impact of the pre-hospital FPS rate on functional outcome should be prospectively studied while controlling for confounding factors.

## Data Availability

The datasets used and/or analysed during the current study are available from the corresponding author on reasonable request.

## Abbreviations

FPS: First-pass success
HEMS: Helicopter emergency medical services
CI: Confidence interval
PHAAM: Prehospital advanced airway management
RSI: Rapid sequence induction
NMBA: Neuromuscular blocking agent
GCS: Glasgow coma score
OHCA: Out-of-hospital cardiac arrest

## References

[1] Rehn M, Hyldmo PK, Magnusson V, Kurola J, Kongstad P, Rognås L (2016). Scandinavian SSAI clinical practice guideline on pre-hospital airway management. Acta Anaesth Scand.

[2] Lockey DJ, Crewdson K, Davies G, Jenkins B, Klein J, Laird C (2017). AAGBI: safer pre-hospital anaesthesia 2017. Anaesthesia.

[3] Chrimes N, Cook TM (2017). Critical airways, critical language. Brit J Anaesth.

[4] Crewdson K, Lockey D, Voelckel W, Temesvari P, Lossius HM, Group EMW (2019). Best practice advice on pre-hospital emergency anaesthesia & advanced airway management. Scand J Trauma Resusc Emerg Medicine..

[5] Mosier JM, Joshi R, Hypes C, Pacheco G, Valenzuela T, Sakles JC (2015). The physiologically difficult airway. West J Emerg Med.

[6] Mosier JM, Sakles JC, Law JA, Brown CA, Brindley PG (2020). Tracheal intubation in the critically Ill. Where we came from and where we should go. Am J Respir Crit Care.

[7] Sklar MC, Detsky ME (2019). Emergent airway management of the critically ill patient: current opinion in critical care. Curr Opin Crit Care.

[8] Sakles JC, Chiu S, Mosier J, Walker C, Stolz U (2013). the importance of first pass success when performing orotracheal intubation in the emergency department. Acad Emerg Med.

[9] Mort TC (2004). The incidence and risk factors for cardiac arrest during emergency tracheal intubation: a justification for incorporating the ASA guidelines in the remote location. J Clin Anesth.

[10] Gellerfors M, Fevang E, Bäckman A, Krüger A, Mikkelsen S, Nurmi J (2018). Pre-hospital advanced airway management by anaesthetist and nurse anaesthetist critical care teams: a prospective observational study of 2028 pre-hospital tracheal intubations. Br J Anaesth.

[11] Ångerman S, Kirves H, Nurmi J (2018). A before-and-after observational study of a protocol for use of the C-MAC videolaryngoscope with a Frova introducer in pre-hospital rapid sequence intubation. Anaesthesia.

[12] Crewdson K, Lockey DJ, Røislien J, Lossius HM, Rehn M (2017). The success of pre-hospital tracheal intubation by different pre-hospital providers: a systematic literature review and meta-analysis. Crit Care.

[13] Yamanaka S, Goldman RD, Goto T, Hayashi H (2020). Multiple intubation attempts in the emergency department and in-hospital mortality: a retrospective observational study. Am J Emerg Med.

[14] Mosier JM (2019). Physiologically difficult airway in critically ill patients: winning the race between haemoglobin desaturation and tracheal intubation. Br J Anaesth.

[15] von Elm E, Altman DG, Egger M, Pocock SJ, Gøtzsche PC, Vandenbroucke JP (2014). The strengthening the reporting of observational studies in epidemiology (STROBE) statement: guidelines for reporting observational studies. Int J Surg.

[16] Saviluoto A, Björkman J, Olkinuora A, Virkkunen I, Kirves H, Setälä P (2020). The first seven years of nationally organized helicopter emergency medical services in Finland – the data from quality registry. Scand J Trauma Resusc Emerg Med.

[17] Saviluoto A, Laukkanen-Nevala P, Raatiniemi L, Jäntti H, Nurmi JO (2021). An analysis of prehospital critical care events and management patterns from 97 539 emergency helicopter medical service missions: a retrospective registry-based study. Eur J Anaesth.

[18] Heino A, Iirola T, Raatiniemi L, Nurmi J, Olkinuora A, Laukkanen-Nevala P (2019). The reliability and accuracy of operational system data in a nationwide helicopter emergency medical services mission database. Bmc Emerg Med.

[19] Sollid SJ, Lockey D, Lossius H (2009). group P advanced airway management expert. A consensus-based template for uniform reporting of data from pre-hospital advanced airway management. Scand J Trauma Resusc Emerg Med.

[20] Sunde GA, Kottmann A, Heltne JK, Sandberg M, Gellerfors M, Krüger A (2018). Standardised data reporting from pre-hospital advanced airway management – a nominal group technique update of the Utstein-style airway template. Scand J Trauma Resusc Emerg Med.

[21] Pakkanen T, Nurmi J, Huhtala H, Silfvast T (2019). Prehospital on-scene anaesthetist treating severe traumatic brain injury patients is associated with lower mortality and better neurological outcome. Scand J Trauma Resusc Emerg Med.

[22] Spaite DW, Hu C, Bobrow BJ, Chikani V, Barnhart B, Gaither JB (2017). The effect of combined out-of-hospital hypotension and hypoxia on mortality in major traumatic brain injury. Ann Emerg Med.

[23] Björkman J, Laukkanen-Nevala P, Olkinuora A, Pulkkinen I, Nurmi J (2021). Short-term and long-term survival in critical patients treated by helicopter emergency medical services in Finland: a registry study of 36 715 patients. BMJ Open.

[24] Björkman J, Setälä P, Pulkkinen I, Raatiniemi L, Nurmi J (2022). Effect of time intervals in critical care provided by helicopter emergency medical services on 30-day survival after trauma. Injury.

[25] Krüger AJ, Lockey D, Kurola J, Bartolomeo SD, Castrén M, Mikkelsen S (2011). A consensus-based template for documenting and reporting in physician-staffed pre-hospital services. Scand J Trauma Resusc Emerg Med.

[26] Abid ES, Miller KA, Monuteaux MC, Nagler J (2022). Association between the number of endotracheal intubation attempts and rates of adverse events in a paediatric emergency department. Emerg Med J.

[27] Kottmann A, Krüger AJ, Sunde GA, Røislien J, Heltne J-K, Carron P-N (2022). Establishing quality indicators for pre-hospital advanced airway management: a modified nominal group technique consensus process. Br J Anaesth.

[28] Olvera D, Patanwala A, Wolfe A, Sakles J (2020). First pass success is important in prehospital tracheal intubation to minimise the risk of physiologic deterioration. Br J Anaesth.

[29] Peters J, van Wageningen B, Hendriks I, Eijk R, Edwards M, Hoogerwerf N (2015). First-pass intubation success rate during rapid sequence induction of prehospital anaesthesia by physicians versus paramedics. Eur J Emerg Med.

[30] Ljungqvist HE, Nurmi JO (2022). Reasons behind failed prehospital intubation attempts while combining C-MAC videolaryngoscope and Frova introducer. Acta Anaesth Scand.

[31] Kim C, Kang HG, Lim TH, Choi BY, Shin Y, Choi HJ (2013). What factors affect the success rate of the first attempt at endotracheal intubation in emergency departments?. Emerg Med J.

[32] Jung W, Kim J (2020). Factors associated with first-pass success of emergency endotracheal intubation. Am J Emerg Med.

[33] Länkimäki S, Spalding M, Saari A, Alahuhta S (2021). Procedural sedation intubation in a paramedic-staffed helicopter emergency medical system in Northern Finland. Air Med J.

[34] Nwanne T, Jarvis J, Barton D, Donnelly JP, Wang HE (2020). Advanced airway management success rates in a national cohort of emergency medical services agencies. Resuscitation.

[35] Hypes C, Sakles J, Joshi R, Greenberg J, Natt B, Malo J (2017). Failure to achieve first attempt success at intubation using video laryngoscopy is associated with increased complications. Intern Emerg Med.

[36] Myatra SN (2021). Airway management in the critically ill. Curr Opin Crit Care.

[37] Pacheco GS, Hurst NB, Patanwala AE, Hypes C, Mosier JM, Sakles JC (2021). First pass success without adverse events is reduced equally with anatomically difficult airways and physiologically difficult airways. West J Emerg Med Integr Emerg Care Popul Heal.

[38] Powell EK, Hinckley WR, Stolz U, Golden AJ, Ventura A, McMullan JT (2019). Predictors of definitive airway sans hypoxia/hypotension on first attempt (DASH-1A) success in traumatically injured patients undergoing prehospital intubation. Prehosp Emerg Care.

[39] Heino A, Laukkanen-Nevala P, Raatiniemi L, Tommila M, Nurmi J, Olkinuora A (2020). Reliability of prehospital patient classification in helicopter emergency medical service missions. Bmc Emerg Med.

[40] Lossius HM, Sollid SJ, Rehn M, Lockey DJ (2011). Revisiting the value of pre-hospital tracheal intubation: an all time systematic literature review extracting the Utstein airway core variables. Crit Care.

[41] Chi JH, Knudson MM, Vassar MJ, McCarthy MC, Shapiro MB, Mallet S (2006). Prehospital hypoxia affects outcome in patients with traumatic brain injury: a prospective multicenter study. J Trauma Inj Infect Crit Care.

